# Efectividad de la crioterapia suministrada por enfermeras para lesiones preneoplásicas del cuello uterino

**DOI:** 10.7705/biomedica.6966

**Published:** 2023-12-29

**Authors:** Edwin Pulido, Mauricio González, Óscar Gamboa, Jairo Bonilla, Joaquín Luna, Raúl Murillo

**Affiliations:** 1 Centro Javeriano de Oncología, Hospital Universitario San Ignacio, Bogotá, D.C., Colombia Hospital Universitario San Ignacio Hospital Universitario San Ignacio Bogotá D.C Bogotá, D.C.; 2 Emeritus, Instituto Nacional de Cancerología, Bogotá, D.C., Colombia Instituto Nacional de Cancerología Instituto Nacional de Cancerología Bogotá D.C Bogotá, D.C.; 3 Servicio de Radioterapia, Instituto Nacional de Cancerología, Bogotá, D.C., Colombia Instituto Nacional de Cancerología Instituto Nacional de Cancerología Bogotá D.C Bogotá, D.C.; 4 Colpolatina SAS, Bogotá, D.C., Colombia Colpolatina SAS Colpolatina SAS Bogotá D.C. Bogotá, D.C.; 5 Colsanitas EPS, Bogotá, D.C., Colombia Colsanitas EPS Colsanitas EPS Bogotá D.C. Bogotá, D.C.; 6 Facultad de Medicina, Pontificia Universidad Javeriana, Bogotá, D.C., Colombia Pontificia Universidad Javeriana Facultad de Medicina Pontificia Universidad Javeriana Bogotá D.C. Bogotá, D.C.

**Keywords:** displasia del cuello del útero, crioterapia, lesiones precancerosas, resultado del tratamiento, Colombia, Uterine cervical dysplasia, cryotherapy, precancerous conditions, treatment outcome, Colombia

## Abstract

**Introducción.:**

El cáncer de cuello uterino es un problema de salud pública relevante en países de ingresos medios y bajos. El seguimiento de mujeres con tamización positiva y el acceso a tratamiento para neoplasia intraepitelial cervical (NIC) son retos mayores en estos países.

**Objetivo.:**

Evaluar la efectividad de la crioterapia suministrada por enfermeras en casos de neoplasia intraepitelial de cérvix.

**Materiales y métodos.:**

Se hizo la inspección visual directa con ácido acético y solución yodada (VIA-VILI), y se practicó colposcopia con biopsia, a mujeres entre los 25 y los 59 años, residentes en zonas de bajos ingresos de Bogotá. Profesionales de enfermería entrenados ofrecieron tratamiento inmediato con crioterapia a mujeres positivas en la inspección visual. Se les practicó colposcopia con biopsia antes del tratamiento y en un control a los 12 meses. Se evaluó la efectividad mediante tasas de curación (resultado: sin lesión) y regresión de NIC2/3 (resultado: ≤NIC1), por verificación colposcópica e histológica.

**Resultados.:**

Se tamizaron 4.957 mujeres. En total, 499 fueron positivas y 472 aceptaron el tratamiento inmediato. Recibieron crioterapia por enfermería 365 mujeres (11 NIC2/3). La tasa de curación fue del 72 % (IC_95%_: 39-94 %) por verificación colposcópica, y del 40 % (IC_95%_: 22-85 %) por histología. Las tasas de regresión fueron del 100 y el 60 %, respectivamente. Se reportaron dos eventos adversos no graves relacionados.

**Conclusiones.:**

Las tasas de curación y regresión por verificación colposcópica son similares a las reportadas con crioterapia administrada por médicos. El tamaño de la muestra con NIC2/3 dificulta la comparación por tipo de verificación. Los hallazgos apoyan la implementación de estrategias de “ver y tratar” por parte de enfermería en poblaciones con acceso limitado a servicios de salud.

## Abstract (en)

**Introduction.:**

Cervical cancer is a relevant public health problem for low- and middleincome countries. Follow-up of positive-screened women and compliance with treatment of precancerous lesions are major challenges for these settings.

**Objective.:**

To evaluate the efficacy of cryotherapy delivered by nurses for cervical intraepithelial neoplasia (CIN).

**Materials and methods.:**

Direct visual inspection with acetic acid and lugol iodine (VIA- VILI), and colposcopy/biopsy were performed on women 25 to 59 years old, residents of low-income areas in Bogotá, Colombia. Trained nurses offered immediate cryotherapy to every woman with positive visual inspection. Colposcopy/biopsy was performed before treatment and at a 12-month follow-up. The effectiveness was measured as cure (outcome: no-lesion) and regression (outcome: CIN1) rates of CIN2/3 using colposcopic and histological verification.

**Results.:**

A group of 4.957 women with VIA/VILI was valuated. In total, 499 were screen positive and 472 accepted immediate treatment. A total of 365 women (11 CIN2/3) received cryotherapy by nurses. Cure rate was 72% (95%CI: 39%-94%) and 40% (95%CI: 22%- 85%) by colposcopic and histological verification, respectively. Regression rates were 100% and 60%. There were two related non-serious adverse events.

**Conclusions.:**

Cure and regression rates by colposcopic verification are like those reported for cryotherapy delivered by doctors. The sample size (CIN2/3) hinders comparisons by type of verification. Our findings support the implementation of screen-and-treat algorithms by nurses among populations with limited access to health services.

En el 2020, el cáncer de cuello uterino fue la cuarta neoplasia maligna con mayor incidencia y mortalidad en las mujeres en el mundo ^1^^,^^2^, y la segunda causa de muerte por cáncer en mujeres de 25 a 39 años ^3^. En Colombia, persiste como una de las neoplasias con mayor incidencia y mortalidad ^3^.

La citología cervical ha sido la principal estrategia de detección temprana del cáncer de cuello uterino ^4^, con la cual se ha logrado reducir la incidencia y mortalidad por esta causa en países desarrollados; sin embargo, en los países de ingresos bajos y medios no ha tenido el impacto esperado debido, entre otras razones, a las múltiples visitas requeridas para el diagnóstico y tratamiento, lo cual genera pérdidas en el seguimiento, empeorado por las barreras de acceso a los servicios de salud ^5^.

Como alternativa para mejorar los programas de detección temprana en regiones con limitado acceso a los servicios de salud, la Organización Mundial de la Salud (OMS) recomienda implementar estrategias de “ver y tratar”, mediante las cuales se llevan a cabo la tamización y el tratamiento de las lesiones precancerosas, en una o dos visitas ^6^. Dicha estrategia ha demostrado reducir la mortalidad y ser costo-efectiva ^7^; no obstante, genera controversia debido al potencial sobretratamiento y, cuando se basa en técnicas de inspección visual, a los problemas de estandarización que afectan la calidad ^8^^,^^9^.

En estas circunstancias, es importante valorar las alternativas que garanticen el acceso a un tratamiento eficaz y seguro. Las estrategias de “ver y tratar” han sido implementadas por distintos proveedores, tanto para el tamizaje (inspección visual) como para el suministro de tratamientos ablativos; se han incluido trabajadores comunitarios, auxiliares y profesionales de enfermería, obstetrices (sic), médicos generales y ginecólogos ^10^. La participación de profesionales de enfermería se ha valorado positivamente en relación con el acceso, la aceptabilidad y la seguridad del tratamiento de lesiones precancerosas, con resultados similares a los reportados con los procedimientos realizados por médicos ^11^. Sin embargo, no encontramos reportes del suministro de tratamiento ablativo por profesionales de enfermería en la región de las Américas, lo que repercute negativamente en su aceptabilidad.

En este estudio, se evalúa la efectividad de la crioterapia suministrada por personal de enfermería a mujeres con lesiones de alto grado, participantes en un programa de ver y tratar basado en la inspección visual directa en Bogotá, Colombia.

## Materiales y métodos

Los métodos del estudio han sido descritos en detalle con anterioridad ^12^. Se incluyeron mujeres entre los 25 y los 59 años con vida sexual iniciada y pertenecientes al régimen subsidiado de aseguramiento del sistema de salud de Colombia (población de bajos recursos y sin capacidad de pago), residentes en tres localidades de bajos ingresos de Bogotá. Se excluyeron quienes estaban en embarazo o con antecedentes de histerectomía, citología vaginal realizada en el último año, procedimientos terapéuticos en el cuello uterino, abortos espontáneos recurrentes, enfermedad física o mental grave, o alergia al ácido acético o al yodo.

Una vez cumplido el proceso de consentimiento informado y evaluados los criterios de elegibilidad, un profesional de enfermería examinaba el cuello uterino mediante especuloscopia. Las mujeres con lesiones sugestivas de cáncer invasor, eran remitidas para tratamiento.

Un profesional de enfermería con entrenamiento específico, practicó la inspección visual directa con ácido acético (VIA) o con lugol (VILI), y un ginecólogo practicó la colposcopia.

Los observadores desconocían los resultados de la valoración realizada por el otro examinador. Los hallazgos se registraron de forma independiente, lo que permitió comparar los resultados.

En el entrenamiento de las enfermeras para la tamización con inspección visual directa y la estandarización de los colposcopistas, se cumplieron las directrices de la *International Agency for Research on Cancer* (IARC) ^13^^,^^14^.

Las pacientes se calificaron como VIA positivas cuando se apreciaban lesiones acetoblancas en contacto con la unión escamocolumnar y, como VILI positivas, cuando había áreas sin captación de yodo (color amarillo) en contacto con la unión escamocolumnar. Los hallazgos colposcópicos se agruparon según el índice de Reid ^15^. A todas las mujeres con clasificación de Reid igual o mayor de 1 en la colposcopia o con VIA o VIA-VILI positivas, se les tomó biopsia guiada por colposcopia de las lesiones identificadas según correspondía. A todas las mujeres con hallazgos positivos, se les practicó de inmediato la crioterapia, en la misma consulta.

Se registraron los resultados de la inspección visual directa (VIA y VILI) y de la colposcopia, y el sitio de toma de la biopsia. Todas las lesiones clasificadas por biopsia como NIC2 o NIC3 que tuvieron reporte de VIA y VILI negativas, se trataron según el protocolo estándar ^14^. Se ofreció tratamiento a las pacientes con lesiones de bajo grado, el cual podía ser inmediato o administrarse en el control de seguimiento, según su persistencia o progresión. El desempeño de las pruebas diagnósticas ha sido reportado previamente ^12^^,^^16^.

El protocolo fue aprobado y supervisado por el Comité de Ética del Instituto Nacional de Cancerología de Colombia.

### Crioterapia

**Figura 1:**
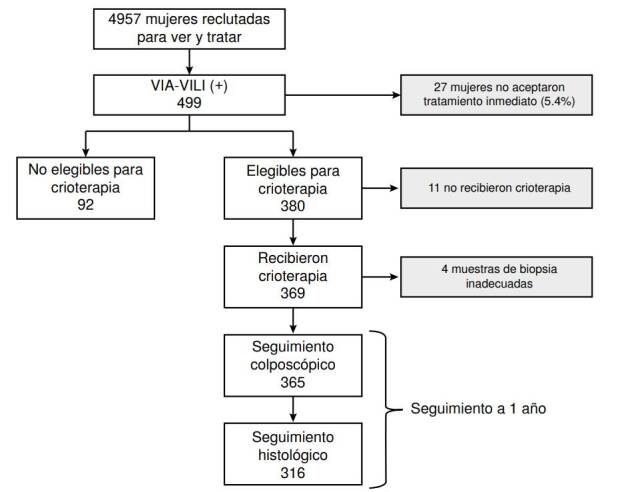
Flujo de pacientes en el estudio

La crioterapia fue administrada por un profesional de enfermería con entrenamiento. El tratamiento se realizó en dos tiempos de tres minutos cada uno con un intervalo de descongelamiento de cinco minutos. Los criterios de elegibilidad para la crioterapia fueron: prueba rápida de embarazo negativa realizada en la misma visita y antes de recibir tratamiento, lesiones localizadas en el exocérvix sin extensión a endocérvix que fueran cubiertas totalmente por la sonda de crioterapia, además, sin evidencia clínica de cáncer. Se entregaron instrucciones para el cuidado postratamiento, e información sobre signos y síntomas de alarma. Quienes no cumplían con los criterios de elegibilidad para crioterapia fueron sometidas a escisión electroquirúrgica con asa (*Loop Electrosurgical Excision Procedure*, LEEP) o remitidas para otro manejo según criterio del colposcopista. El flujo de pacientes se presenta en la figura 1.

### Seguimiento

Se hizo seguimiento telefónico de seguridad a los siete días y, control presencial, a los 15 días. La efectividad se evaluó un año después del tratamiento, con valoración mediante colposcopia a fin de determinar la presencia de alguna lesión residual o complicaciones tardías asociadas. También, se hizo seguimiento telefónico a los seis meses del tratamiento a fin de mantener el cumplimiento de las participantes en el estudio.

Para la evaluación de la lesión residual, se efectuó inspección visual directa con ácido acético y solución yodada (VIA y VILI), y se practicó la colposcopia; también, se tomaron biopsias en las lesiones visibles en la colposcopia. En las mujeres sin evidencia de lesión colposcópica, se tomó biopsia en el sitio de la biopsia original. La presencia de estenosis cervical se evaluó mediante la introducción y rotación de un escobillón de algodón de 5 mm en el canal cervical. La prueba fue interpretada por el ginecólogo.

Quienes presentaron persistencia o progresión de la lesión intraepitelial recibieron tratamiento convencional. Las mujeres con sospecha o diagnóstico de cáncer fueron remitidas para su adecuado manejo.

### Evaluación de efectividad

Se evaluó la efectividad del tratamiento descrito por medio de las tasas de curación y de regresión a una lesión de menor grado en el seguimiento a un año.

La colposcopia y la biopsia fueron los métodos diagnósticos de referencia. La tasa de curación se determinó con base en el protocolo de colposcopia y biopsia. En un primer análisis, se consideró como curación la ausencia de lesión en la colposcopia o la ausencia de lesión en la biopsia de colposcopias positivas; es decir, en este análisis se ignoró el resultado de biopsia sin indicación colposcópica, simulando de esta manera la práctica habitual. Además, se valoró la tasa de curación con base en el resultado de la biopsia, ignorando el resultado de biopsias que no tenían indicación colposcópica; esto fue posible pues hubo pacientes positivas en la inspección visual (VIA y VILI), pero sin indicación colposcópica de biopsia, en quienes, como se indicó previamente, se tomó biopsia de todas formas. La tasa de regresión se calculó de la misma forma, asumiendo como regresión los resultados de NIC1 o menos para las lesiones de alto grado (NIC2/3) en la biopsia inicial. Ambas tasas se reportan como porcentajes con sus respectivos intervalos de confianza.

## Resultados

En total, 4.957 mujeres fueron tamizadas con inspección visual directa (VIA y VILI). De ellas, 499 presentaron prueba positiva y 472 aceptaron recibir tratamiento inmediato, 380 cumplían criterios de elegibilidad para crioterapia según la evaluación realizada por la enfermera y 11 fueron excluidas después de la evaluación colposcópica practicada por el ginecólogo. En total, 369 recibieron tratamiento con crioterapia suministrada por enfermeras, pero 4 se excluyeron del análisis por ser su muestra inadecuada para la biopsia; finalmente, se incluyeron 365 mujeres. El 65,6 % de las mujeres tenía entre 30 y 49 años, el 28,2, entre 25 y 29 años, y el 6,1 %, más de 50 años. Todas las pacientes incluidas cumplieron con la visita de control a los 12 meses.

En el seguimiento colposcópico (sin consideración de biopsias sin indicación colposcópica, se encontró una tasa de regresión de 92 % (IC_95%_: 74-99 %) en mujeres con algún grado de neoplasia intraepitelial inicial. En comparación, todas las mujeres con una lesión NIC2/3 inicial, regresaron a un grado histológico menor. La tasa de curación en mujeres con cualquier grado de NIC inicial fue del 80 % (IC_95%_: 59-93 %) y, en mujeres en cuya biopsia inicial se reportó una lesión de alto grado (NIC2/3), fue del 72 % (IC_95%_: 39-94 %) (cuadro 1).

**Cuadro 1:** Resultados del seguimiento a un año según la valoración colposcópica: si la colposcopia era negativa, se asumía este resultado como el estándar de referencia; si la colposcopia era positiva, se asumía el resultado de histopatología como el estándar de referencia.

Histología de base	n	Seguimiento colposcópico						
		Sin displasia	%	NIC 1 n	%	NIC 2+ n	%	Total
Sin displasia	340	331	97,4	8	2,4	1	0,3	340
NIC1	14	12	85,7	1	7,1	1	7,1	14
NIC2/3	11	8	72,7	3	27,3	0	0,0	11
Total	365	351	96,2	12	3,3	2	0,5	365

NIC: neoplasia intracervical de cérvix

Para el seguimiento histológico (sin consideración de hallazgos colposcópicos), se tuvieron en cuenta los resultados de 316 biopsias. Se encontró una tasa de regresión del 60 % (IC_95%_: 26-88 %) y una tasa de curación del 50 % (IC_95%_: 19-81 %), en mujeres con algún grado de NIC inicial. En aquellas con diagnóstico de NIC2/3 inicial, se reportó una tasa de regresión del 60 % (IC_95%_: 15-95 %) y una tasa de curación del 40 % (IC_95%_: 22-85 % (cuadro 2).

**Cuadro 2:** Resultados del seguimiento histológico a un año, tomando como estándar únicamente el reporte histopatológico, es decir, se ignora el resultado de la colposcopia.

Histología de base	n	Biopsias de colposcopias negativas	Seguimiento histológico						
			Sin displasia n	%	NIC 1 n	%	NIC 2+ n	%	Total
Sin displasia	340	34	211	68,9	95	31,1	0	0,0	306
NIC1	14	9	3	60,0	2	40,0	0	0,0	5
NIC2/3	11	6	2	40,0	1	20,0	2	40,0	5
Total	365	49	216	68,4	98	31,0	2	0,6	316

NIC: neoplasia intracervical de cérvix

## Discusión

Este estudio es el primero en evaluar la efectividad de la crioterapia suministrada por profesionales de enfermería en Colombia y en Latinoamérica, según nuestra revisión.

Se encontró una amplia aceptabilidad de la crioterapia inmediata por parte de las mujeres participantes en el estudio. De 499 mujeres con inspección visual positiva (VIA y VILI), 27 (5,4 %) rechazaron el tratamiento inmediato, lo cual indica una aceptabilidad similar a la reportada en otros estudios ^11^^,^^17^; esto apoya la utilidad de la estrategia de tratamiento inmediato en poblaciones con acceso a los servicios de salud ^10^.

El sobretratamiento ha sido reportado como la principal desventaja de la estrategia de “ver y tratar” por su potencial de morbilidad asociada ^17^; sin embargo, la crioterapia también se propone para tratar la cervicitis crónica. En este estudio, 340 mujeres con histología normal recibieron crioterapia (93,2 % de las mujeres VIA-VILI positivas) y solo se reportaron dos eventos adversos (un caso de enfermedad inflamatoria pélvica y un caso de estenosis cervical), los cuales recibieron manejo ambulatorio sin complicación. Esto equivale a una frecuencia de eventos adversos menor del 0,5 % sin reporte de eventos mayores, lo cual se suma a los hallazgos de diferentes estudios en cuanto al perfil de seguridad de la crioterapia ^11^^,^^17^. El bajo porcentaje de complicaciones asociadas podría justificar su uso en contextos de baja cobertura en salud o en poblaciones de difícil observancia del tratamiento.

En cuanto a la efectividad, se reportaron tasas de curación superiores al 70 % en el seguimiento colposcópico, y del 40 al 50 % por control exclusivamente histopatológico, con tasas de regresión mayores del 90 % por colposcopia y del 60 % por histología. La tasa de curación determinada por colposcopia y biopsia es similar a la reportado previamente. En un estudio en India con tratamiento ablativo suministrado por enfermería, se encontró una tasa de curación de 80 % ^11^, y una revisión sistemática que integra siete experimentos clínicos aleatorizados y 25 reportes de caso mostró tasas de curación del 85 % ^17^.

La principal limitación del presente estudio es la poca cantidad de biopsias analizadas, lo que genera amplios intervalos de confianza y dificulta las comparaciones por el método de verificación. La menor efectividad de la crioterapia evaluada exclusivamente mediante histopatología, en comparación con la evaluación por colposcopia y biopsia, puede estar asociada a factores como la edad y el tipo de zona de transformación, los cuales afectan el rendimiento de la colposcopia ^18^^-^^20^.

De forma general, el desempeño diagnóstico de la colposcopia para detectar lesiones de alto grado, varía ampliamente (sensibilidad del 56,6 % al 80,0 %)^18^^,^^19^. Además, algunos estudios muestran una reducción de la sensibilidad mayor del 10 % en las mujeres mayores de 55 años y una reducción de la sensibilidad cercana al 20 % para las zonas de transformación de tipo 3 comparadas con zonas de transformación de tipo 1 (76,5 % y 92,2 %, respectivamente) ^20^. El análisis detallado de estos factores está fuera del alcance del presente estudio.

Independientemente de los hallazgos con verificación histológica, los resultados basados en la colposcopia con biopsia, es decir, sin considerar las biopsias de colposcopias negativas, se ajustan a la práctica clínica rutinaria, incluyendo el seguimiento de los tratamientos por escisión, por lo que se deben considerar de manera prioritaria los resultados con este tipo de verificación. De hecho, al menos dos pruebas clínicas han demostrado reducción de la mortalidad por cáncer de cuello uterino con el uso de tratamiento ablativo bajo la estrategia de ”ver y tratar” mediante inspección visual o con pruebas para HPV ^7^^,^^21^.

Recientemente, la OMS lanzó una iniciativa para eliminar el cáncer de cuello uterino a nivel mundial, la cual propone alcanzar para el año 2030 elevadas tasas de cobertura de la vacunación contra HPV en menores de 15 años; además, propone lograr una cobertura de tamización del 70 % con una prueba de gran desempeño (pruebas para HPV) con tasas de tratamiento del 90 % de las lesiones identificadas ^22^.

En varias publicaciones se sugiere que uno de los mayores retos de los países de ingresos bajos y medios es el seguimiento de mujeres positivas al tamizaje, incluyendo la confirmación diagnóstica y el tratamiento de la neoplasia intraepitelial de cuello uterino ^23^^,^^24^. Por tal razón, la OMS publicó recientemente una nueva versión de las guías de detección temprana de cáncer de cuello uterino, privilegiando los abordajes de “ver y tratar” o “ver, clasificar (triaje) y tratar”, en los cuales el tratamiento ablativo inmediato es la opción preponderante ^25^.

En este escenario, los profesionales de enfermería con entrenamiento en inspección visual y crioterapia, pueden ser una alternativa ante la poca disponibilidad de médicos especialistas en regiones con acceso limitado a los servicios de salud en Colombia y en Latinoamérica.

A pesar de la toma de decisiones de forma independiente, es posible que, en el presente estudio, la acción de enfermeras y ginecólogos en un mismo espacio haya generado una retroalimentación positiva para el personal de enfermería, favoreciendo así una mejor selección de los casos por tratar con métodos ablativos. Futuros estudios de cohortes permitirían hacer una mejor comparación de resultados entre perfiles, fortaleciendo la evidencia sobre la participación de personal de enfermería.

Con base en nuestros resultados, consideramos que se debe mejorar la oferta educativa a los profesionales de enfermería sobre estrategias de prevención del cáncer de cuello uterino, favoreciendo un rol más participativo en los programas de detección temprana, así como fortalecer el trabajo interdisciplinario con médicos especialistas en ginecología. Una de las mayores inquietudes en torno a los abordajes de ver y tratar, es la elevada variabilidad entre observadores. Sin embargo, en recientes publicaciones, resaltan cómo la estrecha interacción y la permanente retroalimentación entre el personal de cuidado primario (enfermeras) y el personal especializado (ginecólogos), favorece un mejor desempeño con mejoría continua, a la vez de ofrecer mayor acceso y reducir las pérdidas en el seguimiento ^26^.
